# The rise of informed consent and retreat from dependence upon unclaimed bodies in anatomy: An overview and assessment

**DOI:** 10.1002/ase.70171

**Published:** 2025-12-21

**Authors:** David Gareth Jones

**Affiliations:** ^1^ Department of Anatomy University of Otago Dunedin New Zealand

**Keywords:** bequests, ethical values, human tissue acts, informed consent, legislation, retained organs scandals, unclaimed bodies

## Abstract

The development of anatomy has been marked by ethically questionable practices. This has been because the dissection of human bodies has always existed on the periphery of conventional society, necessitating a range of dubious ways of obtaining dead bodies for educational and research purposes. Chief among these has been the use of unclaimed bodies, those obtained without the consent, and on occasion, knowledge of living relatives. This raises the question of the moral status of dead bodies and the place of informed consent in the practice of anatomy. While informed consent has become mandatory in the legislations of many countries, some argue that it is a Westernized concept. The argument here is that informed consent is a crucial ethical requirement for anatomists. By the 1960s legislation in the United Kingdom and similar countries, plus increasing awareness of ethical issues, brought to the fore informed consent and the necessity of using only body donations. This trajectory was strengthened by inquiries into organ retention scandals following postmortems that highlighted the basic ethical values for dealing with the remains of the dead. Nevertheless, there has been continued use of unclaimed bodies internationally, ushering in continued efforts to understand why this is the case and reasons why some indigenous groups reject body donation. It is concluded that except where there are mitigating circumstances, anatomists continuing to rely upon a supply of unclaimed bodies are neglecting the trajectory of ethical debate over the past 40 years.

## INTRODUCTION

Accounts of the development of anatomy as a core biomedical discipline have been well covered by a range of commentators in recent years.^1^, ^2^, ^3^, ^4^ In tracing how human anatomy has developed and changed since the 16th century with the stunningly accurate work of Andreas Vesalius,^5^, ^6^ ethical issues have never been far from the surface although for much of the time they have not occupied a central position. This is largely because attention has been focused on means of obtaining bodies for study. The lack of a ready supply of bodies, let alone the legality of the means employed, has inevitably dominated anatomists' concerns. Consequently, it is their pragmatism that has shone through since, in the absence of bodies for dissection, there would have been no recognizable anatomy today. In Europe in the 18th and 19th centuries, ethical directives came from outside the profession, often in response to public disquiet at what were regarded as disreputable activities and unpalatable behavior, including body snatching, use of the bodies of the neglected and mentally incapacitated, on occasion made worse by deceit and scandal.^7^, ^8^ Even highly respectable figures in medicine and surgery were implicated, either directly or indirectly, in some of these activities, as they advanced their careers in the developing field of anatomy.^9^

From the standpoint of the 21st century, the lack of explicit ethical awareness is striking. While it would be invidious to condemn our forebears for this omission, this history raises questions as to the nature of the trajectory from then to now, with reference to how and when the notion of informed consent became a significant feature. For as long as informed consent was disregarded, it was deemed acceptable for anatomists to routinely use unclaimed bodies for dissection. Once informed consent became a core feature of anatomists' thinking, it appears that ethical best practice is to use only bodies that have been donated. But is there a proviso?

The major thrust of this trajectory has emerged in Western countries where the autonomy of the individual is paramount.^10^ Hence, it may be inappropriate in societies where communal authority holds sway and where the cultural impetus is to protect the bodies of the recently deceased. Consequently, while the impetus is to work toward sole dependence upon donated bodies, it may be unrealistic to impose this upon cultures lacking a Eurocentric approach. Criteria for accepting alternative forms of consent are required if informed consent itself is not to be weakened as a universal model of ethical best practice.

## WHAT IS INFORMED CONSENT?

According to Beauchamp,^11^ the term “informed consent” emerged in the 1950s. Significant discussion of this concept began within medicine, law and philosophy around 1972, which was when it began to highlight the quality of the patient's understanding of the information being provided to them. This in turn allowed the patient to authorize or refuse treatment and/or any biomedical intervention. From Beauchamp's perspective in 2011, the history of informed consent was still unfolding, and that unresolved and critical moral challenges remained. It is not surprising that the same applies to anatomy as to medicine in general.

According to the Declaration of Helsinki^12^ for informed consent to be adequately informed it includes information on the aims and methods of the study, conflicts of interest, and anticipated benefits and risks to those participating. There is to be no coercion on the participants who have a right to withdraw without prejudice at any point. The study should not detrimentally affect the community, and hence consideration should be given to the community and not simply the individual. Voluntariness underlines all aspects of the process.

Informed consent applies in the first instance to the living rather than the dead. This is because it is placed within a cultural context, namely, that based on the will of the individual. It is at home, therefore, within societies in which individual rights and responsibilities predominate rather than in societies where decision‐making is far more communal and where literacy is limited.^13^, ^14^, ^15^ In this sense, it fits far better into Western societies, raising the question of whether it can be applied as rigidly in more communally based societies. However, even in the latter, consent is required as an ethical basis for interacting with one another. Are there then alternatives to fully informed consent?

One alternative is informed proxy consent that is regarded as a way forward for human ancient DNA (aDNA) research where the deceased are represented by living people affected by the research.^10^ Informed consent cannot be applied here due to the lack of living relatives, and proxy consent is seen as analogous to informed consent from living people.^16^, ^17^

The significance of proxy consent in the case of the living is where individuals or communities assume responsibility for decision‐making on behalf of an individual. This does not apply to aDNA research since no consent is possible from the decedent. In this instance, proxy and relational autonomy consent comes into play with a person or group of living people providing permission on behalf of a deceased person.^10^ In terms of aDNA, proxy consent can be obtained from an individual or community on behalf of the deceased.^13^, ^18^ It is an illustration of relational autonomy proxy consent. Permission is obtained for the research from cultural groups who identify with the ancient people under investigation. Rather than diminishing the importance of informed consent, it provides as much consent as possible in the absence of a way to obtain direct informed consent. The aim is to engage as much as possible with communities while being sensitive to the communities' cultural, traditional, and religious practices.^13^

Interesting as is this digression into the varying forms of consent, it appears to be of limited relevance to the consent required for today's dead bodies where there are living relatives, or living relatives who can potentially be contacted. What has emerged is the significance of communities, as opposed to individuals, in societies where they are core to the organization of those societies.

## IS INFORMED CONSENT A WESTERNIZED CONCEPT?

A criticism made of reliance upon notions of informed consent is that it represents a restricted Westernized view that does not represent the breadth of ethical thinking across diverse cultures. To this end, Matisson and Maude^19^ have argued that from an African perspective, unclaimed cadavers should be made available for use in training and research in medical institutions. Based on the work of Metz^20^ and Chemhuru^21^ these authors conclude that we do not have direct duties of respect toward the dead. This is because the dead lack the capacity to enter into social relationships and neither can they have meaningful relationships. Hence, it is argued, unclaimed bodies can be used in training and research.

Matisonn and Muade^19^ acknowledge that there is almost universal insistence on dignified burials or disposal of bodily remains, and that in sub‐Saharan Africa, the deceased are thought to be on a journey into the new world of the dead, necessitating the performance of particular rituals at the time of death.^22^ Hence, the widespread opposition to body bequests. Matisonn and Muade^19^ wish to provide rational grounds for the use of unclaimed bodies based on the notion of moral status. For them something has moral status if it is capable of having a certain casual or intentional connection with another being.^20^ This in turn entails having friendly or loving relationships with others.

Metz^20^ concludes that dead human beings have lost that capacity, and cannot be made better or worse. Consequently, they lack moral status meaning that we do not owe them any direct ethical duties. Chemhuru's^21^ theory of moral status leads to a similar conclusion, namely, that since dead human beings do not have moral status any duty we have toward them is connected to their usefulness to other beings.

The African context, as outlined by Mbiti,^23^ is that although the physical body dies, the spirit or soul persists and escapes to the spiritual realm. On this basis, Matisonn and Muade^19^ conclude that there is no connection between the dead human body and the ancestor. As a result, the body remains purposeless and destroyed. It is from this basis that they wish to see greater use made of dead bodies for use in teaching and research. On these grounds the unclaimed dead should be made available for good uses.

This attempt to move the bar by defining the dead as lacking moral status fails to justify the use of unclaimed bodies. There are a number of reasons for this. According to some scholars dead bodies have the status of quasi persons rather than non‐persons.^24^ Second, indigenous groups have strong beliefs on how the body is to be treated after death, and this is the antithesis of neglecting the body as unclaimed.^25^, ^26^, ^27^, ^28^ Third, it ignores the moral status of the living, that is, the family members and communities of the deceased. Their connection to the deceased and their feelings for the deceased, when alive and now when dead, are not to be ignored.^29^, ^30^, ^31^, ^32^ These reciprocal relationships are ethically crucial in all societies, especially in African societies and other indigenous communities. Fourth, continued use of unclaimed bodies exemplifies the persistence of racial science in human anatomy in a country such as South Africa.^33^ This risks objectifying human remains and perpetuating the marginalization of vulnerable populations through human anatomy.

These considerations suggest that informed consent is not an explicitly Western concept. While not to be applied in a rigid formulaic fashion, some form of consent is crucial. This will vary with cultural expectations and beliefs, leading in some instances to rejection of any interference with the dead body by outside parties, and at the other extreme to openness to body donation. A task awaiting anatomists from diverse cultural backgrounds is to explore how they can best interpret, and implement, informed consent within their own cultural and ideological frameworks. This is because it is difficult to see how any outworking of ethical best practice will lead to the ready availability of unclaimed bodies.

## RETENTION OF BODY PARTS SCANDALS AND INFORMED CONSENT

Moves to recognize the centrality of informed consent are evident in many countries in their legislative requirements governing the treatment of dead bodies, both at postmortem and in the dissecting room. These have largely been driven by external social concerns. Alongside these developments there have been a parallel series of developments—arising from medico‐clinical scandals, of which those in the United Kingdom are the best known. These came to a head around the year 2000, when a number of highly significant reports emerged on the Bristol and Alder Hey scandals.^34^, ^35^ Their significance ethically is that the working parties investigating them emerged with a series of insightful reports spelling out the basic ethical values that need to be considered when dealing with body parts and organs.^36^, ^37^ These apply most forcefully to pathologists, although they are relevant for anatomists.

Organs and body parts had been removed from bodies at postmortem, including coronial autopsies, and retained without consent for indefinite periods of time. The events that became the subjects of these inquiries had largely taken place between the 1960s and 1990s. Significantly this was not a new phenomenon, since brains and hearts had been routinely kept following postmortems in previous eras when informed consent was little emphasized. Pathologists had assumed a right to determine the fate of the material and retention was an end in itself. However, from the 1960s onwards the central role of informed consent was being recognized, and the paternalism of earlier times was being seriously questioned.

The first significant report dealt with events that had transpired in the Bristol Royal Infirmary.^34^ The children in this case had received complex cardiac surgical services between 1984 and 1995. Tissue was routinely taken at or after the postmortem of these children who died following pediatric cardiac surgery. The families of these children were unaware that organs had been retained and had no idea that at the time of the funerals the children's bodies were incomplete. No consent had been sought for this retention. In response, the inquiry proposed that obtaining consent needs to be seen as a process giving the parents adequate time to reflect on how they should respond.

Clear as this was, the outcomes of the inquiry into events at The Royal Liverpool Children's Hospital (Alder Hey) were far more incisive since the events were more extreme.^35^ There had been widespread retention of many organs in addition to those required to determine the cause of death. Additionally, in many instances, the organs had not been used for either teaching or research; they had simply been stored. The public outcry reached a crescendo in 2001 when the findings of the inquiry were released, the activities far surpassing anything that had routinely taken place in the preinformed consent era. A paternalistic approach had reached its zenith and little empathy was shown for the grieving families. The Alder Hey report concluded that a weak and poorly understood legal framework had allowed bad practice to flourish.^35^ Among its recommendations were that the Human Tissue Act 1961 should be amended to insist on fully informed consent for the retention of body parts following postmortem. This led to the 2004 Act in the United Kingdom, with its repercussions for anatomical practice.

These two scandals and ensuing reflections indicated the way in which ethical thinking was developing in the latter part of the 1980s and 1990s. They, alongside complementary reports by government bodies,^36^ ushered in a new set of ethical guiding principles. It has been these that have provided biomedical scientists, including anatomists and pathologists, with a rationale for elevating informed consent to its central role in practice. By proposing specific guidelines, these reports afforded anatomists and others with substantial resources for acting in an ethical manner. Conversely, they left little room for those who continue to utilize unclaimed, that is, unconsented, bodies. By seeking to respond to ethically disreputable practices in the recent past, these guidelines provide a detailed template for current ethical practice. Of the approaches recommended, some are directly applicable for anatomists.^38^ These are:

- *Informed consent* to ensure that those involved are able to exercise their fully informed consent for any donation.
- *Respect*, both for the person who has died and their families.
- *Understanding* the needs of the families whose love and feelings for the person who has died are as strong as they were during life.
- *Cultural competence* signifies the need to be sensitive to the religious and cultural perspectives of different population groups, some of which may be opposed to body donation and any interference with the body after death.
- *A gift relationship* to emphasize the centrality of “donation,” since it is a gift to be received with gratitude.

These principles set the standards for ethical approaches to every aspect of donation, whether of whole bodies, organs, body parts or other tissues, from both the living and the dead. They were the culmination of ethical thinking that had been proceeding from the 1980s onward.

Other reports^37^ emerged with similar ethical frameworks, highlighting the centrality of altruism. This is because it underpins the importance of the community and serving the community's interests. Allied with this is the donors' welfare, with the corollary that no pressure is to be placed on a person or their family to donate. Underlying these strictures are values such as trust and respect; trust that professionals will respect donors' confidentiality, and that donated materials will only be used for purposes for which they were donated. What is evident throughout these reports is a growing maturity concerning ethical understanding and regulation.

From an anatomist's perspective, the advantage of addressing the role of informed consent in this manner is that it does not commence with body donation. The bodies have undergone postmortem examination, and decisions have been rightly taken about the retention of organs and/or body parts to determine the cause of death. The problem arises when there is longer term retention. When this occurs in the absence of any consent, someone is disadvantaged, generally close family members, when they discover at some future time that the body has been buried or cremated minus certain body parts. If family members had not been informed that this was going to happen and had not been provided with an opportunity to provide consent, it may have been assumed that they would have consented had they been informed. This presumed consent cannot be classed as adequate consent since it was not informed and bears the hallmarks of deception and paternalism. There were no cultural pressures militating against informing next of kin, and no attempt was made to involve them in the decision‐making. Consent was by‐passed for no good reason.

The illustrations provided by the retention of body parts/organs scandals all took place within a Western context and where the bodies have known next of kin. These are the families grieving the loss of a loved one where a strong form of informed consent can be expected. The link between the deceased and the living relatives is close. It is in this context that Beauchamp's,^11^ notion that the term “informed consent” emerged in the 1950s is relevant (see “What is Informed Consent?”).

## THE GRADUAL EMERGENCE OF INFORMED CONSENT: A BRITISH PERSPECTIVE

It was during the 1990s that anatomists began to articulate their concerns with continuing use of unclaimed bodies.^39^ The commonalities and differences between issues surrounding the donation of organs for transplantation and the donation of bodies for dissection were noted at that time. Values highlighted by organ donation, namely, the autonomy of the individual, the interests of family members, altruism, and a redemptive element, have parallels with donated, but not unclaimed, bodies. The difference between the two lay in the lack of informed consent and the absence of a gift factor in the case of unclaimed bodies.

A line in the sand was proposed in a 2012 article contending that use of unclaimed bodies amounts to ethically dubious practice.^40^ The reason was that it had opened the profession to a range of questionable ethical practices, from use of the impoverished and mentally ill to stigmatized groups during the Nazi era. Common to all instances was the absence of informed consent. Taken together they reflected negatively on the perception of anatomy and on societies' views of the dead human body. They also underlined a lack of ethical concern on the part of anatomists in their professional deliberations.

It is noteworthy that this article appeared as late as 2012, although the trajectory toward the centrality of informed consent had been well established by this time, as evidenced by changes in United Kingdom legislation. The driving force for the 2004 update in the United Kingdom was the 2000 and 2001 reports of the body parts retention inquiries by pathologists.^34^, ^36^, ^41^ Despite these highly relevant developments, the use of unclaimed bodies by anatomists continued even though, for many in Western societies, they were increasingly regarded as problematic. While the factors involved are diverse, the link between the use of unclaimed bodies and the lack of informed consent had not been made explicit.

The question is whether the use of unclaimed bodies is invariably unethical since some argue that the deceased cannot suffer harms or have their rights violated.^42^, ^43^ This is on the assumption that there are no next of kin and no known individuals with any meaningful interest in the body. If this point is accepted, and it has proved impossible to identify any next of kin or member of a community with direct interest in the body, it may not prima facie be unethical to use an unclaimed body for anatomical purposes as demonstrated by examples in Africa (see below). However, the strictures are substantial, namely, that concerted efforts have failed to identify interested parties in the body.

Acknowledgment of philosophical disagreement on the rights of dead bodies does not justify diminishing the call for informed consent as a normative ethical value. This is because it demeans living relatives and community members where these exist and are potentially contactable.

The development of legislation in the United Kingdom from the 1960s onwards was based on the presumption that informed consent is a legitimate legal and ethical category. The 1961 Act in the United Kingdom emerged as pivotal, since it represented a significant move toward the voluntary donation of bodies and body parts. In the words of the Act, “If any person, either in writing at any time or orally in the presence of two or more witnesses during his last illness, has expressed a request that his body or any specified part of his body be used after his death for therapeutic purposes or for purposes of medical education or research, the person lawfully in possession of his body after his death may, unless he has reason to believe that the request was subsequently withdrawn, authorize the removal from the body of any part or, as the case may be, the specified part, for use in accordance with the request.”^44^

Nevertheless, it was still possible for the person lawfully in charge of the body, such as the medical superintendent of a hospital, to authorize use of the body for anatomical examination, on condition that the deceased person or living relative had not objected. The balance had therefore moved perceptibly toward informed consent albeit with limitations. With hindsight, 1961 stands out as the year of demarcation after which informed consent gradually became recognized as the primary marker of ethical behavior. This denoted the gradual termination of the widespread use of unclaimed bodies as an ethical alternative to bequests, even as it did not bring their use to a complete halt. The ethical tide was turning with an individual's or family's consent becoming central. Over the next few years legislation based on the 1961 United Kingdom Act followed in one country after another.^30^, ^31^, ^32^

Over a period of 40 to 50 years, a revolution in ethical outlook had occurred, commencing in the early 1960s and marked in legislation by this 1961 Act and its heirs elsewhere. This clarified that within United Kingdom jurisdiction, the person lawfully in possession of the body had the statutory authority to approve the removal and use of body parts. It also set out that prior to authorization, this individual had to make as much reasonable inquiry as practicable to ascertain whether any relative objected. This represented a step forward with its recognition that the next of kin have rights marked by the emergence of the phrase “lack of objection.” By today's standards that is ethically weak, but it was far stronger than the previous regime where the family's views were ignored by doctors, pathologists and anatomists on the assumption they had authority to act in ways they considered best. This shift to a “lack of objection” stance represented a significant redirection in control from the professionals to the families.

But “lack of objection” had one major disadvantage, namely, that the family did not have to be informed of the details of the postmortem. As a result, they were often in no position to object. They were unaware of what was being undertaken. It was within this environment that brains were routinely removed at postmortem for use in the teaching of neuroanatomy to medical students. This was valuable educationally; it was a routine procedure and was not considered unethical. With hindsight, its ethical credentials are viewed as impoverished.

The next significant legislative move in the United Kingdom came with the Anatomy Act 1984, according to which individuals who wished their bodies to be used for anatomical dissection had to express this wish, either verbally or in writing.^45^ They also had to be living in the United Kingdom. Following anatomical examination, the body was to be cremated within 3 years of the death of the individual. Responsibility for ensuring compliance with the Act rested with an Inspectorate of Anatomy, a statutory body. The Act insisted that the dead body be used solely for the purpose of exploring and defining topographical anatomy and forbade its use for rehearsing surgical techniques. The Act also regulated the possession of bodies and control of public display. This was fully developed informed consent.

The more recent Human Tissue Act 2004 was enacted in response to concerns raised about the handling of human tissue and organs in the United Kingdom,^46^, ^47^ in the main after the retained organs scandals at Bristol Royal Infirmary and Royal Liverpool Children's Hospital.^34^, ^35^ Its aim is to ensure responsible and ethical practices in the use of human tissue for medical and scientific purposes. It did this by establishing a comprehensive framework for regulating activities involving human tissue by emphasizing the need for informed consent and creating the Human Tissue Authority (HTA) to oversee these activities. The Act mandates informed consent for a range of procedures and makes it illegal to use human tissue for other purposes.

Of particular interest for the present discussion is its emphasis on the need for fully informed consent for the removal, storage, and any use of human tissue, organs, and bodies. Consequently, it regulates activities involving human tissue, namely, postmortem examinations, anatomical studies, research, and transplantation in the United Kingdom. The HTA was created to establish codes of practice and provide guidance on all aspects of human tissue use. The Act prohibits activities without appropriate consent and makes it an offense to use donated bodies or tissue for purposes other than those agreed upon. It also outlines specific purposes for which human tissue can be used, such as research, education, and training. Of major relevance is its prohibition of commercial dealings in human material for transplantation. Some, however, have criticized what they view as a lack of autonomy in any nonconsensual use of organs for transplantation.^48^

With the 2004 Act the move from “lack of objection” to informed consent was completed. Admittedly, this took place in one specific jurisdiction, and a Eurocentric country at that, and so strictly speaking its relevance is limited. However, as in many other instances, the principles driving it are of universal interest and relevance. While not dealing directly with the use of unclaimed bodies in the anatomical world, its concentration on the necessity of informed consent leaves no place for the use of bodies devoid of that level of consent. On the surface, there is no leeway for anatomists to use unconsented bodies when functioning in societies where informed consent is the generally accepted ethical norm within the health sciences.

## CONTINUING USE OF UNCLAIMED BODIES INTERNATIONALLY

However, this ethical norm is far from a universal stance since unclaimed bodies continue to be used in many countries as shown by an overview of current sources of cadavers worldwide.^49^, ^50^ While acknowledging the difficulties of obtaining accurate data of current practices across 165 countries with medical schools, the findings demonstrated that 22 countries exclusively use donated bodies, 21 exclusively use unclaimed bodies, with others using a mix of the two, but with a preponderance of unclaimed bodies. Body donation programs are well established in most parts of Europe and North America, in Australia and New Zealand, and in some parts of Asia and South America. The reasons for continued dependence upon unclaimed bodies vary immensely across different cultures, representing a wide variety of cultural and religious beliefs.^29^, ^51^ Where there are strong objections to body donation local anatomy schools may be faced with two immediate options: giving up dissection altogether, or importing bodies from other countries.^49^, ^52^ This depends upon the existence of an international trade of dead bodies that itself raises many ethical queries.^53^ Other options based on use of computer programs, online resources, 3D models, virtual reality and augmented reality may not be available to those in financially constrained settings.

In spite of these disparities in practice, international anatomical associations such as the International Federation of Associations of Anatomy (IFAA),^54^ the American Medical Association (AMA),^55^ the American Association of Clinical Anatomists (AACA),^56^ and the Trans‐European Pedagogic Anatomical Research Group (TEPARG)^57^ have concluded that dependence upon unclaimed bodies in present‐day anatomy teaching breeches core ethical standards. Approval of next of kin emerges as central to body acquisition. This demonstrates that serious ethical reflection, advocacy and practical support are required if a donation paradigm is to be adopted in these countries. But it leaves unanswered the continuing viability of the use of unclaimed bodies and whether such usage is as ethically problematic as suggested by these international organizations.

### United States

Unfortunately, the ongoing use of unclaimed bodies is not limited to countries lacking a historical background in ethical debate on the ethical superiority of donations. The worldwide study of body donations classes the United States as “mostly body donation,”^49^, ^53^, ^58^ a surprising categorization when the United Kingdom, for example, is “exclusively body donation.”^59^, ^60^

In 2019, about 12.4% of United States anatomy schools used unclaimed bodies.^61^ A detailed investigation into the situation in Texas found that during 2017–2021 six of 14 medical schools directly obtained and possibly used unclaimed bodies or used unclaimed bodies transferred from other schools.^62^ Another eight schools did not use unclaimed bodies. Of particular interest was the rise in the number of unclaimed bodies from 2.27% in 2017 to 14.12% in 2021. The immediate conclusion to draw from this move is that decisions are being taken to utilize unclaimed bodies in preference to donations in the face of well documented arguments that informed consent is a core ethical prerequisite for accepting bodies. Reliance upon unclaimed bodies is going counter to guidelines set by the IFAA and the AMA, guidelines that serve as the basis for anatomical policies on dissection and anatomical training.

These decisions are being taken against the background of considerable local publicity about the indignity of using the unclaimed bodies of the poor in Texas and the ways in which some of these have ended up unclaimed.^63^ The lack of informed consent has allowed a repetition of the many unsavory episodes that blighted the history of anatomy. Overall trends are that the unclaimed bodies are disproportionately black, male, mentally ill, and homeless; family members cannot be easily reached, or cannot or will not pay for cremation or burial.^63^ Even more disturbing, some of the bodies were supplied to for‐profit medical training and medical technology companies, on the grounds that this converted a tragic situation into one of hope through advancing medical research. This was no consolation for those families who had been searching for missing relatives. Those involved in the use and distribution of unclaimed bodies are riding roughshod over any notion of informed consent, thereby undermining the ethical foundations of medical education and practice. The lack of respect shown to family members, leading to their remorse and anguish at being unable to help their loved ones at the time of their death, is the antithesis of ethical medical practice.

In addressing these data Shupe^64^ has argued that it is ethically impermissible for anatomy schools to accept unclaimed bodies on the basis of lack of consent, harm to the deceased, the unjust nature of the practice, and its undermining of public trust in medicine and medical professionals. Together they constitute a coherent series of ethical concerns ranging from an overriding of the autonomy of, and lack of respect for, those affected, a lower ethical standard than applies to organ donation, and providing substandard care to vulnerable groups within society. Even in the absence of federal regulations banning the use of unclaimed bodies, whole body donation programs have ethical obligations to ensure that they only accept donated bodies,^53^, ^58^ and implement their own regulations. No doubt there are barriers including financial ones in some situations, but this serves to reiterate the tension regularly encountered between what can be achieved ethically within social and financial constraints. This is an ongoing challenge, but substantial ethical resources are now available within the anatomical community to ensure an increasingly ethical course worldwide.

### Africa

An important example is provided by South Africa that has progressively transitioned to greater reliance upon donated bodies from dependence largely upon the use of unclaimed bodies.^65^, ^66^, ^67^, ^68^ This move has been driven by rejection of the ethical acceptability of unclaimed bodies, even though legislation in South Africa accepts the use of unclaimed bodies by government designated authorities to medical institutions.^69^ It makes provision for the donation of bodies by individuals other than the donor themselves. This is generally donation by a family member of a relative's body without the consent of the deceased, on condition that the deceased had not stipulated during life an unwillingness to have their body donated after death. The reasons are frequently financial and high funeral costs,^70^ and use of unclaimed bodies is surrounded by restrictions.

South African commentators, such as Billings and co‐workers,^67^ regard this legislation as a reflection of a dubiously ethical sociohistorical practice even as they seek to transform it. Nevertheless, they are aware of the factors militating against body donations by traditional African religions and belief in ancestors,^28^, ^29^, ^71^ and the significance of rituals around burials,^72^, ^73^ and burial customs.^28^ Reluctance of black South Africans to donate their remains is, in part, a legacy of apartheid, and this together with various spirituality and burial practices, leads to the situation whereby White bodies make up the bulk of the donor programs.^67^

The South African illustration is of a society seeking to come to terms with cultural and political legacies, in which the predominant pattern was usage of unclaimed bodies. The Anatomy authorities are committed to the ethical superiority of donated bodies and most of the medical schools are moving in that direction. By the same token they do not downplay the legitimate cultural objections of some indigenous groups, while holding up the IFAA principles as their ethical baseline.

Also within the South African context is its National Health Act No 61 of 2003 and its inspectors of anatomy. A survey of the small number of inspectors revealed how they deal with pauper burials and the ethical issues raised by these.^74^ The National Health Act has provision for the use of unclaimed indigent bodies by Anatomy schools. The body of an unclaimed and unidentified individual is to be stored in a freezer within 7 days of death, and if unidentified after 30 days the local municipality is to provide a pauper burial or cremation of the body. It allows for a spouse, family member or friend to claim the body but if that does not occur the body passes to the health officer and inspector of anatomy. The onus therefore is on the claiming of bodies if possible. It is only when this does not eventuate that it passes to an Anatomy school for use in teaching and/or research.

While these South African examples err on the side of using donated rather than unclaimed bodies, some deem this to be a Western‐centric approach.^19^, ^23^ There is no doubt that the primary thrust of debate within anatomical circles has accepted the move toward reliance on donated bodies.^29^, ^67^ This is in spite of acknowledgment that many cultures in Africa reject the possibility of donation.^28^ In these cultures, the question of informed consent does not arise since the possibility of body donation will not arise.

This is helpfully shown by a study of West African universities where many of the anatomy programs rely on the use of unclaimed and unidentified bodies, frequently abandoned in hospitals or mortuaries.^51^ Against this bleak background, concerted efforts are required to build body donation programs, to identify deceased persons through outreach services, and to support students psychologically when confronted by bodies killed in abhorrent ways. Respectful handling is an essential ethical imperative for establishing the integrity of the discipline under these far from ideal conditions.^51^

## CONCLUDING COMMENTS

Except where there are mitigating circumstances, it is disturbing that sections of the anatomical world continue to be reliant upon unclaimed bodies with their lack of any form of consent, let alone informed consent. It signifies acceptance of ethical standards that have been queried for at least 40 years. What then of Vesalius and the room for maneuver given him for acting “unethically” in his time and culture? Does the same apply to contemporary anatomists in countries with neither a history of ethical anatomical practice nor legislation to undergird such practice? Are they in a position parallel to that of Vesalius in the 16th century? Sympathetic as the anatomical community should be toward anatomists in this situation, Vesalius does not provide a satisfactory precedent. He was a researcher mapping out details of human anatomy in an environment largely averse to the anatomical concepts he was enunciating for the first time. He was moving into unmapped territory following novel educational and philosophical concepts. Additionally, he had no alternatives for obtaining bodies. Today, societies with limited previous knowledge of anatomy teaching exist in a world where these concepts have been elaborated and well disseminated internationally. Nevertheless, difficulties remain in some quarters that encounter resource constraints, limited access to global networks, and on occasion political instability. Institutions in these situations may not be willfully noncompliant. What they need is the support of the international anatomical community via education, moral support, and practical assistance. This is an ongoing challenge for those who have benefited from decades of ethical discernment, and with the resources to implement best practice.

The argument has recently been put forward that the discipline of anatomy is to be nontransgressive.^75^ By this, the authors mean that it is not to go beyond acceptable boundaries of taste and decorum. They contend that the dissection of human bodies obtained through a donor program need not be transgressive if due respect is accorded the donated bodies and if the students are ethically prepared. By these expectations, the practices of Vesalius would be classed as transgressive, but that demonstrates how far anatomy has come over the intervening years, and also how far anatomy in some countries still has to go.

Almost hidden in this debate is another reality in countries like South Africa with a predominant black population but where the donor population consists mainly of older White individuals. This imbalance means that students are unable to study a representative sample of bodies, anatomically and of the population they will encounter in clinical practice. It also has limitations for research. This is an unintended consequence of reliance upon donation and rejection of the use of unclaimed bodies. While this is a topic for future investigation, it raises question of what constitutes transgressive anatomy in societies where body donation is limited to one subset of their cultural groups.

The ethical environment in which anatomists have functioned over their long history has been in transition for many years, during which distinctly different ethical periods can be detected.^5^, ^9^, ^76^ Each period contributed to some aspect of anatomical understanding, and each operated within an ethically compromised setting. They represent different ethical worlds, ranging from one where there was no alternative to obtaining bodies for research purposes without consent to others where there was flagrant abuse of the most basic of moral precepts. Over these periods, ethical expectations on how human beings are to be treated in both life and death were being transformed within many societies, with implications for anatomical communities.

Some of the historical practices would be anathema today, but in their own era and with the state of anatomical ignorance at the time, they present a different perspective. This does not justify everything they did, and we must be prepared to draw boundaries around their actions and strive today for better ethical practices.

Throughout the 1980s and 1990s there was increasing awareness of the need to take next of kin into consideration. Two illustrations are helpful here. The first has nothing to do with dead bodies, but points to the need for open communication with patients and the importance of informed consent. This was the 1988 Cartwright Inquiry in New Zealand, when experimental treatment had been undertaken on patients in the absence of providing them with relevant information, let alone their informed consent.^77^ The second was a 1995 report by the Nuffield Council of Bioethics in the United Kingdom on human tissue.^78^ This was relatively general in nature with its focus on ethical issues that were coming to light internationally across the biomedical sciences in the 1980s and 1990s. It emphasized the importance of respect for the dead body in clinical medicine and in medical research.

Different lines of evidence have led to the conclusion that informed consent, and hence body donation, should constitute the guiding ethical standard for anatomists. Two years likely represent the outer boundaries of the transition within a British context—1961 (United Kingdom legislation) and 2000 (when the reports following the British organ scandals became widely known). By the 1980s, anatomists were in a position to reasonably recognize the questionable morality of continuing to rely on unclaimed bodies, given the ethical resources at their disposal (regulation and legislation, professional guidance, bioethical frameworks in which informed consent is central, and growing public sentiment against the practice). Subsequent to the 1980s, anatomists continuing to use unclaimed bodies can reasonably be held to be ethically negligent as a general standard.

What then of atrocities prior to the 1960s when informed consent was not generally taken into account? The Nazi atrocities in the 1930s and 1940s stand as grim examples. The procedures were so egregious that their unethical nature stands out apart from any reference to informed consent. The anatomists who benefited from a ready supply of unconsented unclaimed bodies were morally complicit in mass murder.^76^ In other words, important as informed consent is it is not the only ethical barometer; murder is murder.

## LIMITATIONS

The arguments running through this review have concentrated on examples from the United Kingdom, in terms of the legislation quoted and the organ retention scandals. While they do not represent all situations in every country, they have served as models for legislation in many other societies, as demonstrated by the ongoing transformation in attitudes and practice in South Africa. Since the organ retention scandals were investigated very thoroughly, they revealed the fundamental ethical values relevant to ways of treating the dead body and the families of the deceased. Hence, it is not unreasonable to argue that this starting point in the United Kingdom has widespread application. There is a great deal still to be done in African and Asian contexts. Except for South Africa, these have not been referred to in detail here, and far more meticulous attention needs to be given to them to ascertain the forms of consent best suited to their cultural milieus.

## AUTHOR CONTRIBUTIONS

**David Gareth Jones:** Conceptualization; writing – original draft; methodology; writing – review and editing.
